# Efficacy and safety of first-line targeted and immunotherapy for metastatic colorectal cancer: a network meta-analysis

**DOI:** 10.3389/fimmu.2025.1643133

**Published:** 2025-11-04

**Authors:** Liman Huo, Hongyu Yue, Ruixia Yang, Xiaoli Sun, Ziyue Wang, Hong Liu, Jiang Liu, Rui Feng, Ping Liang

**Affiliations:** Department of Pharmacy, Fourth Hospital of Hebei Medical University, Shijiazhuang, China

**Keywords:** metastatic colorectal cancer, first-line therapy, targeted drugs, immunotherapy, network meta-analysis

## Abstract

**Background:**

As targeted therapies and immunotherapy become increasingly prevalent in treating metastatic colorectal cancer (mCRC), comparative analyses are essential to determine the most effective and safe treatment combinations. This study aims to compare and rank the efficacy and safety profiles of first-line systemic treatments for mCRC.

**Methods:**

This network meta-analysis was conducted in compliance with PRISMA guidelines, reviewing randomized controlled trials from PubMed, Embase, Web of Science, Cochrane Library, and ClinicalTrials.gov through March 2024. A network meta-analysis is conducted using a Bayesian random effects mode. After the data was extracted, data analysis was conducted in gemtc R. The primary outcomes measured were overall survival (OS), progression-free survival (PFS), and the incidence of adverse events (AEs) graded ≥3.The Cochrane risk-of-bias assessment tool was used to evaluate the quality of each study.

**Results:**

A total of 61 RCTs involving 20,579 patients were included. The results showed that FOLFOXIRI combined with bevacizumab and atezolizumab significantly improved PFS and OS, with HRs for PFS and OS of (HR:0.19, 95% CI: 0.11–0.33), (HR:0.48, 95% CI: 0.30–0.78), respectively. The incidence of ≥ Grade 3 AEs was high, but no new fatal treatment-related AEs were observed, and the safety of this regimen was manageable. FOLFOXIRI in combination with anti-EGFR monoclonal antibody regimens showed significant PFS and OS improvements in the RAS/BRAF wild-type subgroup. For the subgroup of patients aged ≥ 70 years, thetrifluorouridine-tipiracil plus bevacizumab regimen also had some advantage in PFS and OS. Although the incidence of Grade ≥ 3 AEs was higher, the incidence of AEs was similar across age groups and well tolerated in this regimen, and it was more suitable for elderly cancer patients.

**Discussion:**

These findings underscore the importance of integrating targeted drugs and immunotherapy in first-line mCRC treatments, highlighting significant differences in efficacy and safety profiles that can guide therapeutic decisions.

**Systematic Review Registration:**

https://www.crd.york.ac.uk/PROSPERO/view/CRD42024604107, identifier CRD42024604107.

## 1 Introduction

Colorectal cancer (CRC) ranks as the third most common malignancy globally, with projections indicating approximately 1.9 million new cases and 904,000 deaths in 2022. Accounting for about 10% of all cancer cases and deaths, CRC thus represents the second leading cause of cancer-related mortality (1). Reflecting substantial heterogeneity among CRC subtypes, approximately 20% of CRC patients present with synchronous metastases at diagnosis, and an additional 50% develop metastases as the disease progresses (2). For isolated metastases, surgical and local ablation techniques can be effective; however, systemic therapies remain crucial for advanced stages due to issues like non-selective tumor targeting and resistance to chemotherapy drugs, which result in a five-year survival rate of only 10% to 30% for these patients (3).

According to NCCN guidelines, the primary first-line treatment for CRC primarily relies on oxaliplatin or irinotecan as the basis for monotherapy (FULV and XEL), two-drug combination therapies (FOLFOX, FOLFIRI, and XELOX), and three-drug combination therapies (FOLFOXIRI). In recent years, significant progress has been made in targeted therapy and immunotherapy. When combined with chemotherapy, these treatments have notably enhanced the treatment response and survival rate of metastatic colorectal cancer (mCRC) (4). The primary molecular targets for mCRC therapy encompass EGFR, RAS, BRAF, VEGF, and HER2. The FDA has approved numerous drugs targeting these pathways, including cetuximab, panitumumab, bevacizumab, and regorafenib. Immunotherapy drugs primarily consist of nivolumab and Pembrolizumab (5).The effectiveness of these targeted therapies or immunotherapies used in conjunction with chemotherapy often depends on the mutant status of the target, MSI/MMR status, and the location of the primary tumor (left or right) (6). Through the integration of targeted therapy and immunotherapy, the treatment landscape for mCRC has undergone significant changes. This study aims to assess the efficacy and safety of first-line treatment options for mCRC, with the objective of providing a comprehensive ranking to assist clinical decision-making based on both efficacy and safety.

Unlike previous network meta-analyses (NMAs) (7–11), which mainly evaluated advantages of immunotherapy combined with targeted as well as chemotherapy, This study also performed a subgroup analysis of patients with mCRC, with special attention to individuals aged 70 and older, this study applied NMA methodology to compare chemotherapy alone or in combination with targeted therapy or immunotherapy by integrating results from 61 first-line treatment clinical trials for mCRC (12).

## 2 Methods

### 2.1 Search strategy

This study was conducted in accordance with the Preferred Reporting Items for Systematic Reviews and Meta-Analyses (PRISMA) guidelines (13). It was registered in PROSPERO with a registration number of CRD42024604107.We performed systematic searches of PubMed, Embase, Web of Science, Cochrane Library, and ClinicalTrials.gov up to March 31, 2024, focusing exclusively on clinical trials involving human subjects. Additional sources were identified through bibliographic reviews of relevant articles. Searches were restricted to English-language publications. Keywords included “colorectal neoplasms,” “metastatic,” “targeted therapy,” “immunotherapy,” “chemotherapy”, and “first-line treatment.” Details of the search strategy are outlined below.

### 2.2 Inclusion and exclusion criteria

Inclusion criteria: 1. Study type: phase II/III randomized controlled clinical trials (RCTs) focusing on first-line treatment for mCRC. 2. Study subjects: individuals with confirmed diagnosis of mCRC. 3. Intervention: the study must incorporate well-defined first-line treatment protocols, encompassing trials involving chemotherapy, targeted therapy, and immunotherapy. 4. Outcome indicators for reporting: the study must report at least one significant outcome indicator pertaining to therapeutic effectiveness, safety, or survival, including overall survival (OS), progression-free survival (PFS), and the incidence of adverse events (AEs). Exclusion criteria: 1. non-RCTs, including observational studies, case reports, reviews, etc. 2. Subjects who do not meet the diagnostic criteria for mCRC or have other severe comorbidities that could potentially impact the study outcomes. 3. Interventions that lack clarity or fail to align with the definition of first-line treatment. 4. Studies with incomplete data or an inability to extract critical outcome indicators. 5. For studies published repeatedly, only the most comprehensive or the most recent version will be considered. 6. Articles not published in English.

### 2.3 Literature screening and data extraction

Data extraction was independently performed by two reviewers (HY Y and RX Y), with disagreements resolved by a third reviewer (P L). Extracted information included the first author’s name, publication year, trial number, pathological type, interventions, sample size, and participant demographics (age and gender).

### 2.4 Statistical methods

Outcome measures for time-to-event variables used hazard ratios (HR), and odds ratios (OR) were used for effect sizes, with estimates deemed significant if 95% credible intervals did not include 1 (14). Data synthesis used a random effects model, modified for NMA settings with initial settings of 20,000 pre-iterations and 100,000 iterations. Statistical analyses were performed using the gemtc R package (15), employing a Bayesian framework that integrates both direct and indirect evidence. Model convergence was verified using 50,000 MCMC iterations and the node-splitting method for consistency checks between direct and indirect evidence, while heterogeneity was assessed using I^2^ values (14). The effectiveness of each treatment was assessed through the surface under the cumulative ranking curve (SUCRA), which ranges from 0% to 100%. A higher SUCRA score signifies superior ranking in terms of efficacy or safety outcomes.

### 2.5 Quality assessment

The quality of included studies was assessed using the Cochrane Risk of Bias Assessment Tool (16). This assessment covers seven domains (1): Random sequence generation (selection bias); (2) Allocation concealment (selection bias); (3) Blinding of researchers and participants (performance bias); (4) Blinding of outcome assessment (detection bias); (5) Completeness of outcome data (attrition bias); (6) Selective reporting (reporting bias); and (7) Other biases not mentioned above. According to the assessment criteria, each item is categorized as “low risk”, “high risk”, or “unclear risk”. The risk of bias assessment graph visually represents these categories using different colors.

## 3 Results

### 3.1 Literature search outcomes

**Figure 1:**
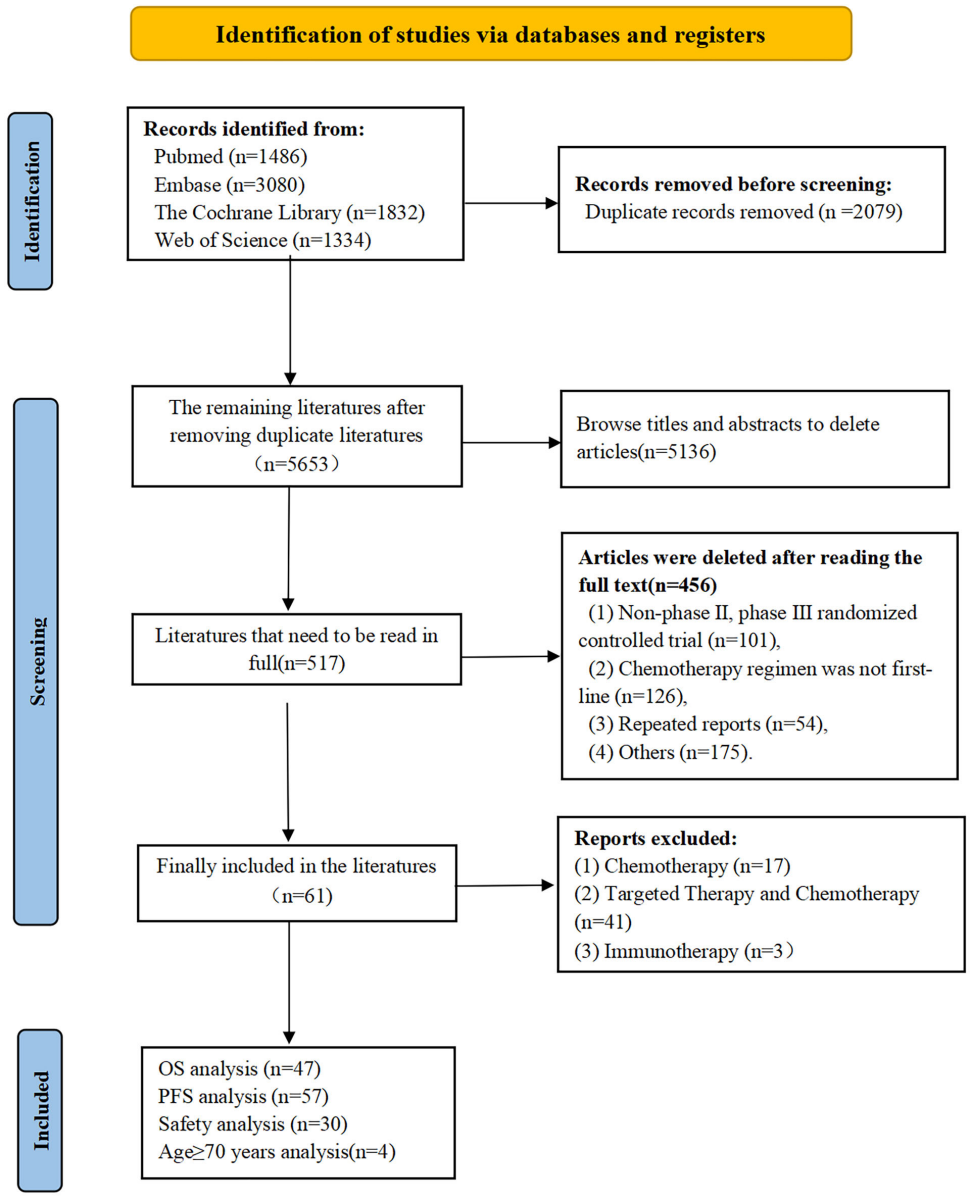
Literature search flowchart.

From our searches we retrieved 5,653 articles, narrowed down to 517 potentially relevant articles after preliminary screening. Rigorous review and adherence to inclusion and exclusion criteria led to 61 RCTs being selected for detailed analysis (**Figure 1**).

### 3.2 Study characteristics

We analyzed 61 phase II/III RCTs, including two three-arm trials, involving a total of 20,579 participants. A total of 30 treatment regimens were included, with the majority receiving FOLFOX, FOLFIRI, or FOLFOXIRI chemotherapy either alone or in combination with targeted therapies such as bevacizumab, cetuximab, or panitumumab. Immunotherapy was limited to four clinical trials: FOLFOX + nivolumab(N), FOLFOXIRI + bevacizumab(B) + atezolizumab(A), pembrolizumab(P), and FOLFOX + avelumab + AdCEA vaccine (17–77). Furthermore, we performed subgroup analyses stratified by RAS/BRAF mutation status and age. Sixteen RAS/BRAF wild-type RCTs involving 4,812 participants were analyzed, encompassing 13 treatment regimens combining chemotherapy with bevacizumab, cetuximab, or panitumumab. Additionally, two RCTs involving 348 RAS/BRAF mutant mCRC cases and incorporating four treatment regimens were analyzed. For patients aged 70 years or older, a subgroup analysis was conducted on four RCTs with 1,498 participants, encompassing five treatment regimens: FULV, XEL, FULV +B, XEL + B, and trifluridine-tipiracil +B. The essential characteristics of the studies included are presented in **Table 1**.

**Table 1:** Basic characteristics of the included studies.

The First Author	Year of publication	Research Name	Phase	gene expression type	Treatment Regimen			Sample size				Gender			Age (Year)		
					Option 1/Experimental Group	Option 2/control group	Option 3/control group	Total	Option 1	Option 2	Option 3	Option 1	Option 2	Option 3	Option 1	Option 2	Option 3
Giacchetti (13)	2000	/	III		FOLFOX	FULV		200	100	100		66/34	64/36		61	61	
Douillard (41)	2000	/	III		FOLFIRI	FULV		387	198	187		132/66	99/88		62	59	
Kabbinavar (19)	2003	/	II		FULV+B	FULV		104	35 33	36		17/18 15/18	27/9				
Hurwitz (20)	2004	/	III		FOLFIRI+B	FOLFIRI		813	402	411		237/165	248/16		60	59	
Kabbinavar (21)	2005	/	II		FULV+B	FULV		209	104	105		56/44	51/49		71I	71	
Kohne (22)	2005	EORTC40986	III		FOLFIRI	FULV		430	214	216		136/78	132/84		61	60	
Comella (23)	2005	/	III		FOLFOX	FOLFIRI		276	140	136		81/59	72/64		62	62	
Colucci (24)	2005	/	III		FOLFOX	FOLFIRI		360	182	178		109/73	93/85		62	62	
Kalofonos (25)	2005	/	II		FOLFOX	FOLFIRI		295	148	147		92/56	90/57		65	66	
Martoni (26)	2006	/	II		XELOX	FOLFOX		118		56		33/29	28/28		67	64	
Souglakos (27)	2006	/	III		FOLFOXIRI	FOLFIRI		283	137	146		76/61	82/61		66	66	
Goldbergl (28)	2006	/	III		FOLFOX	FOLFIRI		305	154	151		90/64	97/54		58	60	
Hospers (29)	2006	/	III		FOLFOX	FULV		302	151	151		100/51	88/63		62	62	
Diaz-Rubio (30)	2007	/	III		XELOX	FOLFOX		342	171	171		107/64	100/71		64	65	
Falcone (31)	2007	/	III		FOLFOXIRI	FOLFIRI		244	122	122		75/47	69/53		62	64	
Hochster (32)	2008	TREE	II		FOLFOX+B FOLFOX+B XELOX+B	FOLFOX FOLFOX XELOX		120 120 120	71 70 72	49 50 48		61/39 49/51 58/42	57/43 62/38 65/35		64 57 62	62 62 63	
Ocvirk (33)	2010	CECOG trial	II	KRAS wt	FOLFIRI+C	FOLFOX+C		62	28	34		17/11	22/12		64	63	
Tebbutt (34)	2010	MAX study	III		XELOX+B	XELOX		313	157	156		102/55	98/58		67	69	
Bokemeyer (35)	2011	OPUS	II	KRAS wt	FOLFOX+C	FOLFOX		179	82	97		42/40	55/42		62	59	
Van Cutsem (36)	2011	NCT00154102	III	KRAS wt	FOLFIRI+C	FOLFIRI		666	316	350		196/120	211/13		61	59	
Cassidy (37)	2011	NO16966	III		XELOX ± B	FOLFOX ± B		2034	1017	1017		612/405	595/41		61	60	
Ducreux (38)	2011	ML169	III		XELOX	FOLFOX		306	156	150		100/56	90/60		66	64	
Guan ZZ (39)	2011	ARTIST	III		FOLFIRI+B	FOLFIRI		203	139	64		70/69	36/28		53	50	
Tveit (40)	2012	NORDIC-VII Study	III	KRAS/ BRAF mutation	FOLFOX+C	FOLFOX		379	194	185		120/74	100/85		61	61	
Cunningham (41)	2013	AVEX	III		XELOX+B	XELOX		280	140	140		84/56	84/56		76	77	
Douillard (42)	2014	PRIME	III	KRAS wt	FOLFOX+P	FOLFOX		656	325	331		217/108	204/12		62	61	
Schwartzberg (43)	2014	PEAK	II	KRAS wt	FOLFOX+P	FOLFOX+B		285	142	143		86/56	96/47		63	61	
Heinemann (43)	2014	NCT00433927	III	KRAS wt	FOLFIRI+C	FOLFIRI+B		592	297	295		214/83	196/99		64	65	
Cremolini (44)	2015	TRIBE study	III	RAS/ BRAFwt	FOLFOXIRI+B	FOLFIRI+B		508	252	256		150/102	156/10		61	60	
Gruenberger (45)	2015	NCT00778102	II		FOLFOXIRI+B	FOLFOX+B		80	41	39		29/12	18/21		63	57	
Carrato (46)	2017	NCT00885885	II	KRAS/ RAS wt	FOLFIRI+P	FOLFOX+P		77	39	38		28/11	31/7		63	65	
Hurwitz (47)	2018	NCT01765582	II		FOLFOXIRI+B	FOLFOX+B		280	185	95		103/82	59/36		56	58	
Sebastian S (48)	2023	AIO KRK0116	II	BRAFV600E Mutation	FOLFOXIRI+C	FOLFOXIRI+B		107	72	35		40/32	14/21		62	64	
Antoniotti, C (49).	2022	NCT03721653	II		FOLFOXIRI+B+A	FOLFOXIRI+B		218	145	73		83/62	42/31		60	61	
Bendell, J. C (50)	2019	NCT02141295	II		FOLFOX+ Vanucizumab	FOLFOX+B		189	95	94		38/57	56/38		63	64	
Cremolini, C (52)	2020	NCT02339116	III		FOLFOXIRI+B	FOLFOX6+B FOLFOX+B		679	339	340		181/158	206/134		60	61	
Denda, T (53)	2021	UMIN-CTR:000007834	III		SIR+B	XELOX+B		484	241	243		151/90	143/100		64	65	
Diaz, L. A (54)	2022	KEYNOTE- 177	III	dMMR/ MSI-H	Pembrolizumab	FOLFOX6 ± B/C FOLFIRI ± B/C		307	153	154		71/62	82/72		63	62.5	
Goldberg, R. M (55)	2023	/	II		FOLFOX	IROX	IFL	795	264	267	264	161/103	157/110	172/92	61	61	61
Khalil, K. A (56).	2022	NCT05316818	II		FOLFOXIRI	FOLFIRI/ FOLFOX		64	32	32		14/18	15/17		42.5	50	
Maiello, E (57).	2020	GOIM 2802	II		XELOX+B	FOLFOX+B		132	87	45		46/41	22/23		66	62	
Modest, D. P (58)	2019	NCT01328171	II	RAS wt	FOLFOXIRI+P	FOLFOXIRI		96	63	33		41/22	24/9		58	60	
Nishizawa, Y (59)	2021	UMIN000006706	II	KRAS wt	SOX+B	SOX+C		45	22	23		14/8	15/8		67	66	
Oki, E (60)	2019	NCT01836653	II	RAS/RAS wt	FOLFOX+B	FOLFOX+C		129	64	65		34/23	34/25		64	65	
Parikh, A. R (61)	2019	MAVERICC	II		FOLFOX+B	FOLFIRI+B		376	188	188		122/66	117/71		61	61	
Redman, J. M (62)	2022	/	II		FOLFOX+B	FOLFOX+Avelumab+AdCEAVaccine		20	10	10		3/7	6/4		——	——	
Sadahiro, S (63)	2020	00001464	II		SIRI+B	FOLFIRI+B		98	51	47		33/18	28/19		65	64	
Tang, W	2020	NCT01972490	II	RAS Mutation	FOLFOX6+B	FOLFOX6		241	121	120		79/42	80/40		58	59	
Watanabe, J (64)	2023	NCT02394795	III		FOLFOX6+P	FOLFOX+B		802	400	402		252/148	268/134		66	66	
André T (65)	2023	NCT03869892	III		Trifluridine-Tipiracil+B	capecitabine+B		856	426	430		240/186	226/204		73	73	
de Gramont (66)	2023	/	III		FOLFOX	FULV		420	210	210		127/83	122/88		63	63	
Tournigand (67)	2023	GERCOR Study	III		FOLFIRI	FOLFOX		220	109	111		62/47	80/31		61	65	
Van Cutsem E (68)	2020	NCT02743221	II		Trifluridine-Tipiracil+B	XELOX+B		153	77	76		40/37	48/28		73	75.5	
Heinemann V (69)	2021	NCT00433927	III	RAS wt	FOLFIRI+C	FOLFIRI+B		400	199	201		52/147	68/133		64	64	
Hu H (70)	2021	NCT02063529	II	RAS/BRAFwt	FOLFOXIRI+C	FOLFOXIRI		101	67	34		58/9	29/5		52	55	
Aranda E (71)	2020	NCT01640405	III		FOLFOXIRI +B	FOLFOX+B		349	172	177		118/54	119/58		61	59	
Ten Hoorn, S (72).	2023	CAIRO2	III	RAS/BRAF V600E wt	XELOX+B+C	XELOX+B		273	142	131		–	–		63	63.8	
Schmoll, H. J (73).	2024	AIO CHARTA	II		FOLFOXIRI+B	FOLFOX+B		242	121	121		79/42	78/43		62	60	
Qin, S (74).	2023	TAILOR	III	RAS wt	FOLFOX+C	FOLFOX		308	146	162		99/47	118/44		56	57	
Meltzer, S (75).	2022	NCT03388190	II	MSS/pMMR(RAS/BRAF+/-)	FOLFOX+ Nivolumab	FOLFOX		76	38	38		18/20	23/15		61	65	
Rossini, D (75).4	2022	NCT03231722	III	RAS/BRAFwt	FOLFOXIRI+P	FOLFOX+P		435	218	217		136/82	138/79		59	59	

### 3.3 Risk of bias

**Figure 2:**
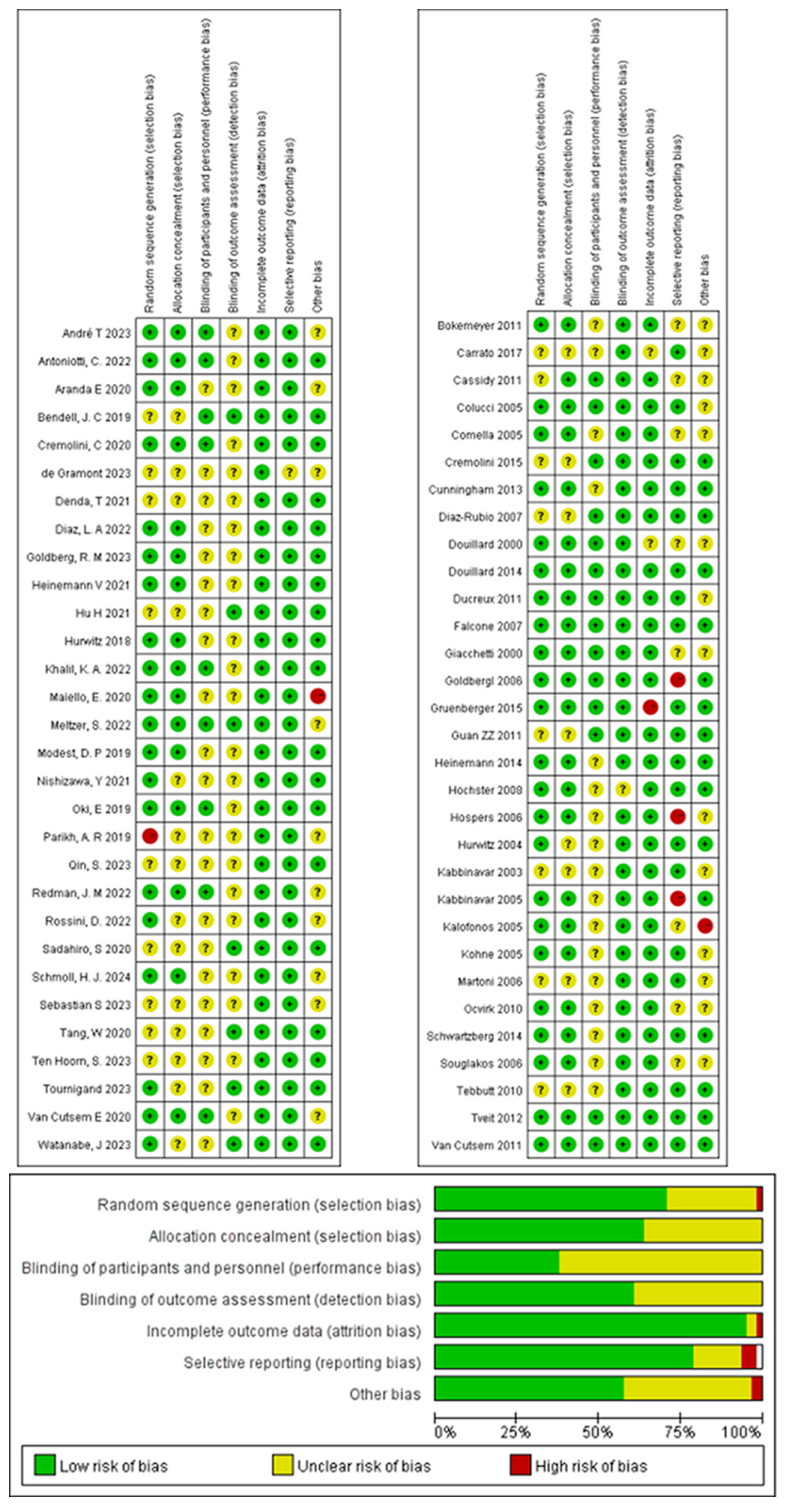
Offset risk assessment.

The risk of bias analysis revealed no specific concerns, except for a potential selection bias for allocation concealment, due to the absence of detailed information on this aspect in all of the RCTs. The bias was assessed using Review Manager 5.3.2 to confirm the medium and high quality of all the included studies (**Figure 2**).

### 3.4 Network evidence mapping for each intervention

**Figure 3:**
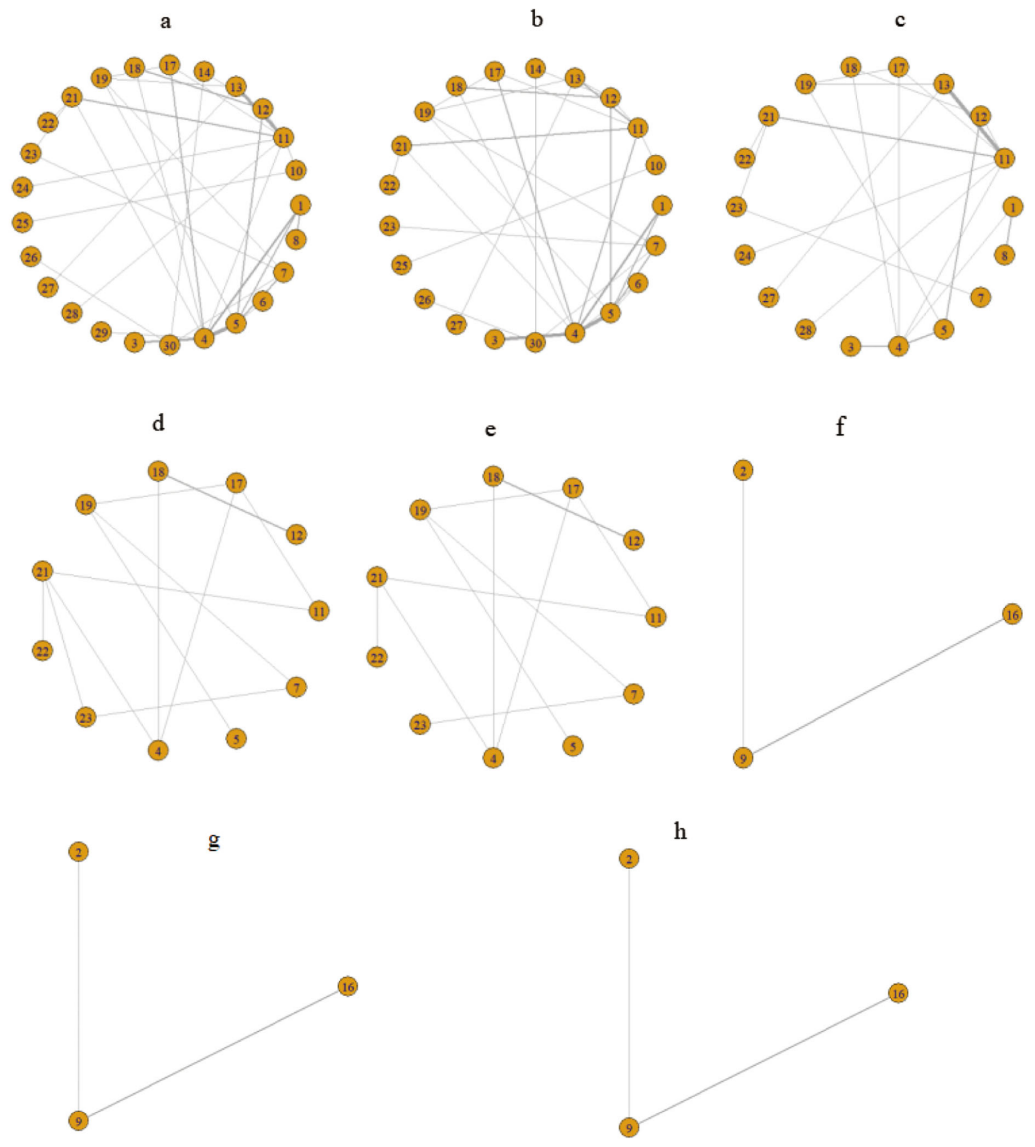
Network relationship diagram of outcome indicators. **(A)** Network evidence diagram for PFS; **(B)** Network evidence diagram for OS; **(C)** Network evidence diagram for grade ≥3 AEs; **(D)** Network evidence diagram for PFS in the RAS/BRAF wild-type subgroup; **(E)** Network evidence diagram for OS in the RAS/BRAF wild-type subgroup; **(F)** Network evidence diagram for PFS in the subgroup of patients aged ≥70 years; **(G)** Network evidence diagram for OS in the subgroup of patients aged ≥70 years; **(H)** Network evidence diagram for grade ≥3 AEs in the subpopulation aged ≥70 years. 1: FULV, 2: XEL, 3: XELOX, 4: FOLFOX, 5: FOLFIRI, 6: IROX, 7: FOLFOXIRI, 8: FULV + bevacizumab(B), 9: XEL + bevacizumab(B), 10: XELOX + bevacizumab(B), 11: FOLFOX + bevacizumab(B), 12: FOLFIRI + bevacizumab(B), 13: FOLFOXIRI + bevacizumab(B), 14: SIRI + bevacizumab(B), 15: SOX + bevacizumab(B), 16: Trifluridine-tipiracil + bevacizumab(B), 17: FOLFOX + cetuximab(C), 18: FOLFIRI + cetuximab(C), 19: FOLFOXIRI + cetuximab(C), 20: SOX + cetuximab(C), 21: FOLFOX + panitumumab(P), 22: FOLFIRI + panitumumab(P), 23: FOLFOXIRI + panitumumab(P), 24: FOLFOX + vanucizumab, 25: XELOX + bevacizumab(B) + cetuximab(C), 26: Pembrolizumab(P), 27: FOLFOXIRI + bevacizumab(B) + atezolizumab(A), 28: FOLFOX + avelumab + AdCEA vaccine, 29: FOLFOX + nivolumab(N), 30: the treatment protocol for the control group consisted of FOLFOX6 ± B/C and FOLFIRI ± B/C.

This NMA encompassed a comprehensive assessment of 30 first-line drug interventions. **Figure 3** illustrates the intricate interconnections among various first-line therapeutic measures. The yellow spheres signify individual interventions, with the accompanying numbers denoting their respective treatment plan codes. The lines connecting these spheres represent direct comparisons between two interventions, and the thickness of these lines is indicative of the number of studies comparing the two measures. After excluding studies with inadequate outcome data and those unable to be integrated into the network, we delved into the PFS of 25 treatment regimens across 57 trials (**Figure 3A**), the OS of 21 treatment regimens in 47 trials (**Figure 3B**), and the occurrence of grade ≥3 AEs associated with 18 treatment regimens in 30 trials (**Figure 3C**).

Furthermore, we conducted a subgroup analysis specific to the RAS/BRAF wild-type, evaluating the PFS and OS of 11 treatment regimens in 15 clinical trials (illustrated in **Figures 3D, E**, respectively). Additionally, for patients aged ≥70 years, we performed a subgroup analysis to assess the PFS, OS, and grade ≥3 AEs of three treatment regimens in three clinical trials (depicted in **Figures 3F–H**, respectively).

### 3.5 Model convergence and inconsistency

The trajectory map reveals that each chain exhibits an overlapping model, which poses challenges in visually identifying individual chains during the iterative process. The density figure demonstrates a distribution curve that closely resembles the normal distribution, with all bandwidth values converging towards stability and tending to zero. Additionally, the Brooks-Gelman-Rubin diagnosis figure indicates that both the median and 97.5% reduction factor tend to approach unity, while a PSRF value of 1.00 indicates complete convergence. Consequently, it can be concluded that the model exhibits excellent convergence.

### 3.6 Results of heterogeneity and inconsistency testing

The node analysis method examines the consistency of selected comparative outcomes. The node analysis graph indicates that the P-values for direct, indirect, and network comparisons of PFS, OS, and grades 3 or higher AEs are all greater than 0.05, indicating no statistical difference and strong consistency. The I^2^ test revealed heterogeneity in OS, PFS, and grades 3 or higher AEs, with some I^2^ values exceeding 50%. Consequently, we employed a random effects model for analysis.

### 3.7 Survival analysis

#### 3.7.1 Survival analysis of all patients

##### 3.7.1.1 Progression-free survival

**Figure 4:**
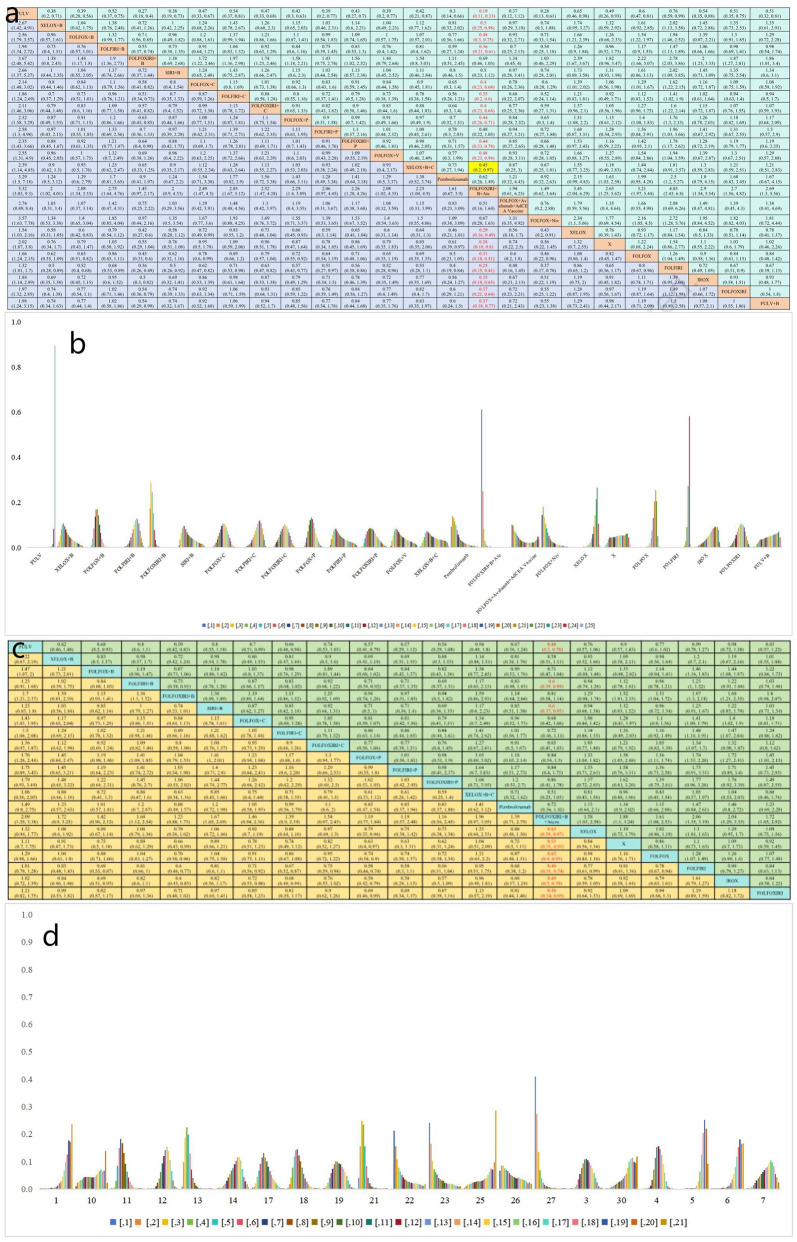
**(a)** Forest plot of PFS; **(b)** Rank probability of PFS; **(c)** Forest plot of OS; **(d)** Rank probability of OS.

In terms of PFS, 25 treatment groups encompassing 16,031 patients were analyzed (**Figure 3A**). The combination of targeted therapy or immunotherapy with chemotherapy demonstrated superior PFS outcomes. The ranking probabilities of 25 treatment regimens were presented in a histogram, with FOLFOXIRI + B + A topping the list with a probability of 0.61. Statistically significant differences were observed between FOLFOXIRI + B + A (HR=0.19, 95% CI 0.11-0.33) and other regimens such as FULV (HR=0.38, 95% CI 0.18-0.8), XELOX (HR=0.31, 95% CI 0.18-0.51), FOLFOX (HR=0.25, 95% CI 0.15-0.41), FOLFIRI (HR=0.35, 95% CI 0.18-0.65), IROX (HR=0.37, 95% CI 0.21-0.64), FOLFOXIRI (HR=0.37, 95% CI 0.18-0.77), FULV + B (HR=0.5, 95% CI 0.25-0.98), XELOX + B (HR=0.48, 95% CI 0.3-0.75), FOLFOX + B (HR=0.36, 95% CI 0.21-0.61), FOLFIRI + B (HR=0.4, 95% CI 0.23-0.68), FOLFOX + cetuximab(C) (HR=0.35, 95% CI 0.2-0.6), FOLFIRI + C (HR=0.4, 95% CI 0.23-0.68), FOLFOXIRI + C (HR=0.44, 95% CI 0.26-0.71), FOLFOX + P (HR=0.44, 95% CI 0.23-0.78), FOLFOXIRI + P (HR=0.48, 95% CI 0.23-0.98), FOLFOX + V (HR=0.45, 95% CI 0.2-0.97), and XELOX + B + C (HR=0.29, 95% CI 0.16-0.49) (**Figure 4A**). In turn, FOLFOXIRI + B+ A demonstrated increased, albeit not significantly different, PFS benefits compared to FOLFOXIRI + B [HR=0.69, 95% CI 0.46-1.03), SIRI + B(HR=0.5, 95% CI 0.23-1.12), FOLFOX + avelumab + AdCEA vaccine (HR=0.51, 95% CI 0.16-1.64), and FOLFOX + N (HR=0.67, 95% CI 0.28-1.63) (**Figure 4A**). In conclusion, FOLFOXIRI + B+ A is recommended as the preferred first-line treatment for mCRC in terms of PFS (**Figure 4B**).

##### 3.7.1.2 Overall survival

OS analysis was conducted on a cohort of 18,128 patients, evaluating 21 different treatment regimens (**Figure 3B**). Statistical analysis indicated a significant difference in OS for FOLFOXIRI + B+ A compared to FULV (HR=0.48, 95% CI 0.3-0.78), FOLFIRI + B (HR=0.6, 95% CI 0.39-0.89), SIRI + B (HR=0.6, 95% CI 0.37-0.95), XELOX (HR=0.63, 95% CI 0.39-0.97), FOLFOX (HR=0.62, 95% CI 0.40-0.93), FOLFIRI (HR=0.49, 95% CI 0.31-0.74), IROX (HR=0.4, 95% CI 0.31-78), and FOLFOXIRI (HR=0.58, 95% CI 0.34-0.95). FOLFOXIRI + B + A demonstrated in turn greater, but not significantly different, OS benefits compared to XELOX + B (HR=0.58, 95% CI 0.31-1.11), FOLFOX + B (HR=0.71, 95% CI 0.47-1.04), FOLFOXIRI + B (HR=0.81, 95% CI 0.58-1.14), FOLFOX + C (HR=0.68, 95% CI 0.42-1.06), FOLFIRI + C (HR=0.72, 95% CI 0.46-1.11), FOLFOXIRI + C (HR=0.65, 95% CI 0.41-1.03), FOLFOX + P (HR=0.84, 95% CI 0.54-1.3), FOLFIRI + P (HR=0.84, 95% CI 0.4- 1.75), FOLFOXIRI + P (HR=0.86, 95% CI 0.41-1.78), XELOX + B + C (HR=0.51, 95% CI 0.25-1.03), and pembrolizumab (HR=0.72, 95% CI 0.36-1.41) (**Figure 4C**). In **Figure 4D**, the bar graph illustrates the ranking probability of these regimens for OS, with FOLFOXIRI + B+ A having a probability of 0.41 for ranking first. In summary, FOLFOXIRI + B+ A is recommended as the first-line treatment for mCRC in terms of OS.

#### 3.7.2 Subgroup survival analysis

**Figure 5:**
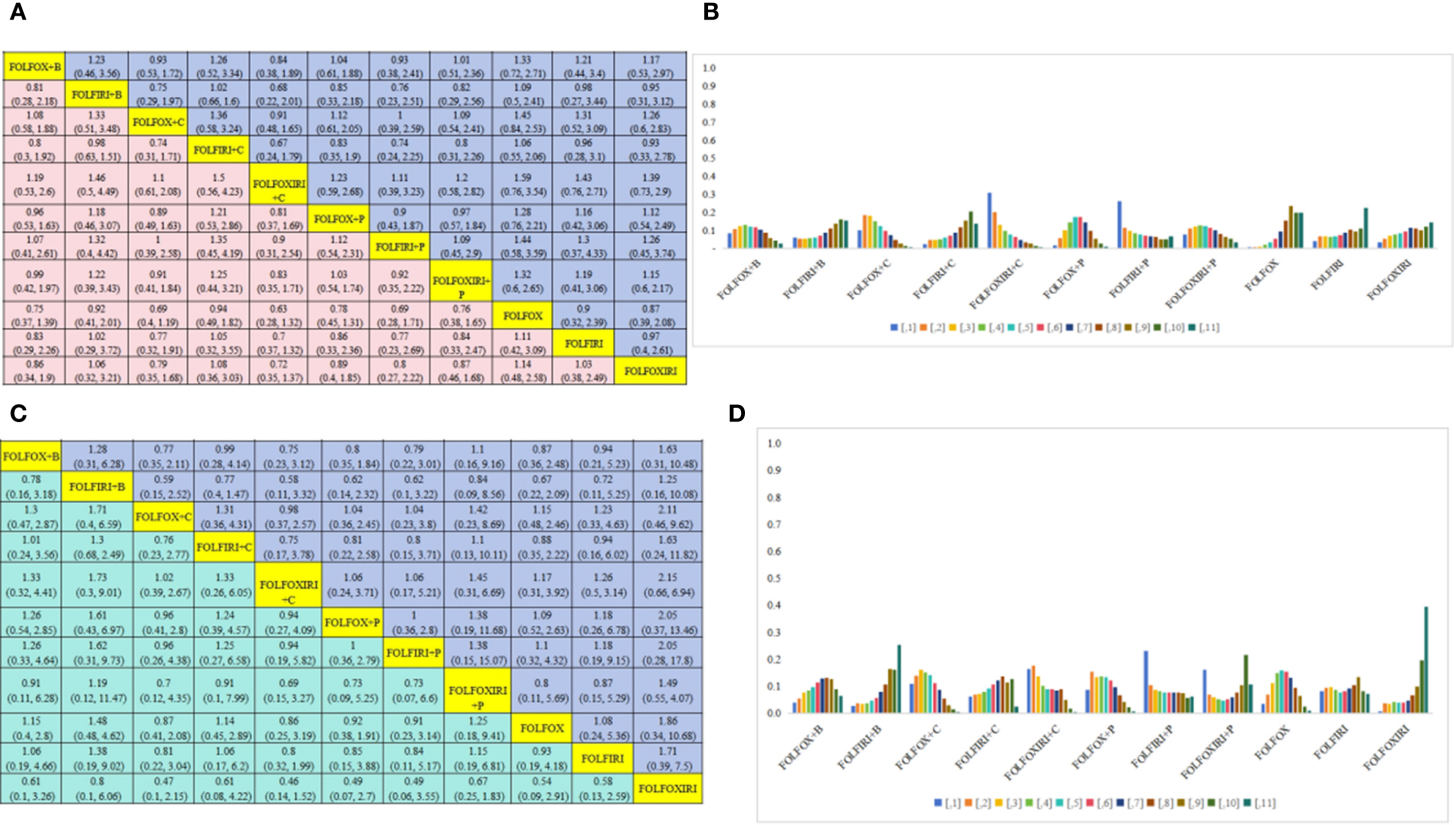
RAS/BRAF wild-type subgroup analysis. **(A)** Forest plot of PFS; **(B)** Rank probability of PFS; **(C)** Forest plot of OS; **(D)** Rank probability of OS.

Further PFS analysis was conducted on a total of 4,812 patients who belonged to the RAS/BRAF wild-type subgroup. This analysis evaluated seven distinct treatment regimens (**Figure 3D**). Statistical analysis revealed that FOLFOXIRI + C demonstrated a superior PFS benefit compared to FOLFOX + B(HR=0.84, 95% CI 0.38-1.89), FOLFIRI + B (HR=0.68, 95% CI 0.22-2.01), FOLFOX + C (HR=0.91, 95% CI 0.48-1.65), FOLFIRI + C (HR=0.67, 95% CI 0.24-1.79), FOLFOX + P (HR=0.81, 95% CI 0.37-1.69), FOLFIRI + P (HR=0.90, 95% CI 0.31-2.54), FOLFOXIRI + P (HR=0.83, 95% CI 0.35-1.71), FOLFOX (HR=0.63, 95% CI 0.28-1.32), FOLFIRI (HR=0.70, 95% CI 0.37-1.32), and FOLFOXIRI (HR=0.72, 95% CI 0.35-1.37); however, the differences failed to reach statistical significance. In conclusion, FOLFOXIRI + C is highly recommended as the frontline therapeutic approach for enhancing PFS among patients with mCRC belonging to the RAS/BRAF wild-type subgroup (**Figure 5A**). **Figure 5B** presents a bar graph illustrating the ranking probabilities of these regimens for PFS within the RAS/BRAF wild-type subgroup. Among these regimens, FOLFOXIRI + C emerged as the most favorable choice with a probability of 0.31.

OS analysis was next conducted on 4,377 patients belonging to the RAS/BRAF wild-type subgroup, assessing 11 distinct treatment regimens **Figure 3E**). Statistical analysis revealed that FOLFIRI + P exhibited greater OS benefits compared to FOLFOX + B (HR=0.79, 95% CI 0.22-3.01), FOLFIRI + B (HR=0.62, 95% CI 0.1-3.22), FOLFOX + C (HR=1.04, 95% CI 0.23-3.8), FOLFIRI + C (HR=0.8, 95% CI 0.15-3.71), FOLFOXIRI + C (HR=1.06, 95% CI 0.17-5.21), FOLFOX + P (HR=1, 95% CI 0.36-2.8), FOLFOXIRI + P (HR=0.73, 95% CI 0.07-6.6), FOLFOX (HR=0.91, 95% CI 03-3.14), FOLFIRI (HR=0.84, 95% CI 0.1-5.17), and FOLFOXIRI (HR=0.49, 95% CI 0.06-3.55); however, the difference failed to reach statistical significance. In conclusion, FOLFIRI + P is recommended as the preferred first-line treatment for enhancing OS in patients with mCRC belonging to the RAS/BRAF wild-type subgroup (**Figure 5C**). **Figure 5D** presents a bar graph depicting the ranking probabilities of these regimens for OS in the RAS/BRAF wild-type subgroup. FOLFIRI+P emerged as the top-ranked treatment, with a probability of 0.23.

An additional analysis was conducted on 348 patients belonging to the RAS/BRAF mutant subgroup, involving two clinical trials and four treatment regimens: FOLFOXIRI + C, FOLFOXIRI + B, FOLFOX + B, and FOLFOX. However, due to the inability to establish a network, this data could not be utilized for network analysis.

**Figure 6:**
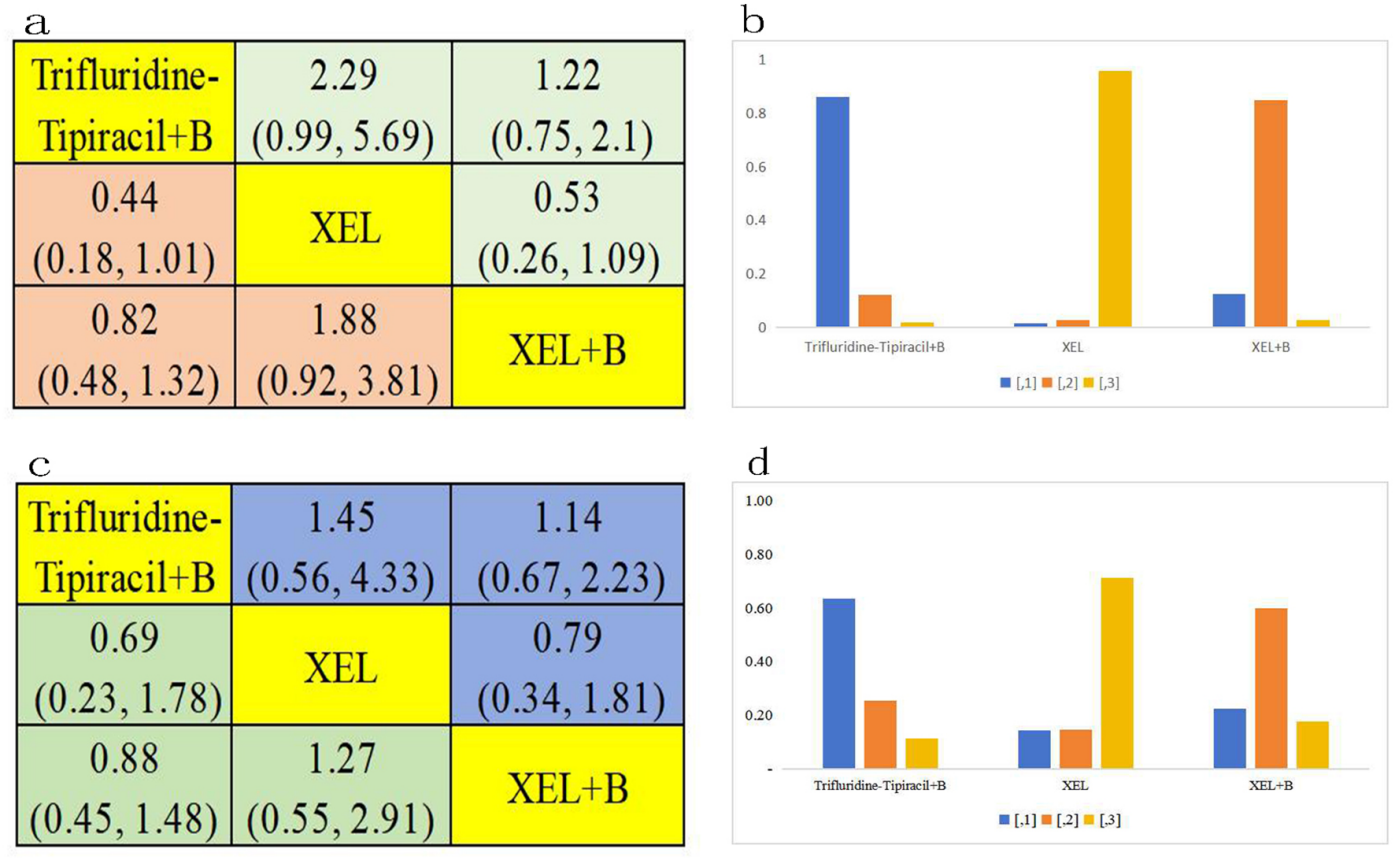
Age ≥70 years subgroup analysis. **(A)** Forest plot of PFS; **(B)** Rank probability of PFS; **(C)** Forest plot of OS; **(D)** Rank probability of OS.

Subgroup PFS analysis was next performed on all 1289 mCRC patients aged over 70 years, using data from three clinical trials and three treatment regimens, namely trifluridine-tipiracil + B, XEL + B, and XEL (**Figure 3F**). Statistical analysis showed that trifluridine-tipiracil + B exhibits greater, albeit not significantly different, PFS benefits in relation to XEL + B(HR=0.82, 95% CI 0.48-1.32) and XEL (HR=0.44, 95% CI 0.18-1.01) (**Figure 6A**). The bar graph in **Figure 6B** illustrates the ranking probabilities of these regimens for PFS in this subgroup. Trifluridine-tipiracil + Branked first, with a probability of 0.63. In conclusion, trifluridine-tipiracil + B is recommended as the frontline therapy for enhancing PFS in mCRC patients over 70 years old.

OS analysis was also conducted among the 1289 patients aged over 70 years, encompassing three clinical trials and three distinct treatment regimens: trifluridine-tipiracil + B, XEL + B, and XEL (**Figure 3G**). This regimen demonstrated also superior, although not significantly different, OS benefits compared to XEL + B(HR=0.88, 95% CI 0.45-1.48) and XEL (HR=0.69, 95% CI 0.23-1.78) (**Figure 6C**). **Figure 6D** presents a bar graph depicting the ranking probabilities of these regimens for OS in this patient subgroup. Once again, trifluridine-tipiracil + B emerged as the top-ranked treatment, with a probability of 0.63. Thus, trifluridine-tipiracil + B is recommended as the preferred first-line treatment option for enhancing OS in mCRC patients over 70 years of age.

### 3.8 Safety outcomes

#### 3.8.1 Grade ≥3 AEs

**Figure 7:**
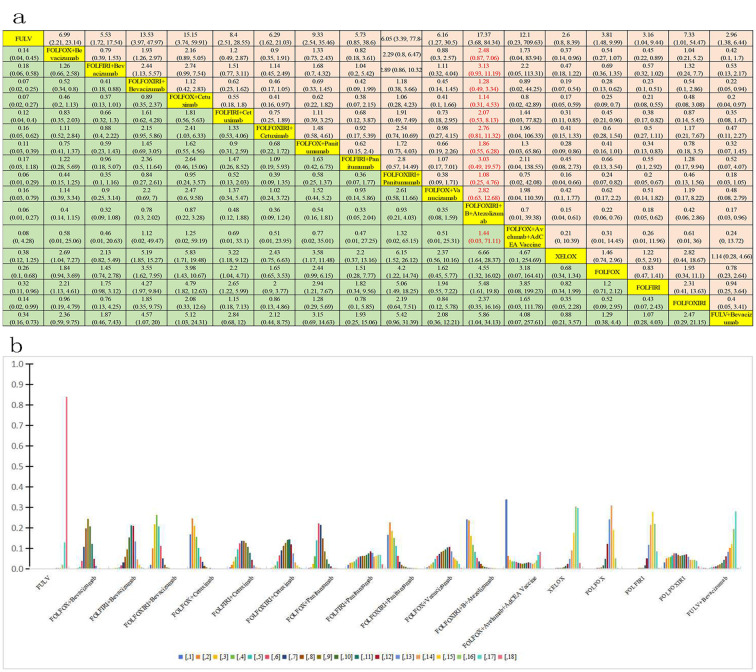
Incidence of grade ≥3 AEs. **(A)** Forest plot of total grade ≥3 AEs; **(B)** Rank probability of total grade ≥3 AEs.

Further analysis was conducted to assess the incidence of grade ≥3 AEs among 11,014 patients, evaluating 18 distinct treatment regimens (**Figure 3E**). **Figure 7** presents a bar graph depicting the ranking probabilities of these regimens based on the occurrence of grade ≥ 3AEs. The combination of FOLFOX + avelumab + AdCEA vaccine emerged as the top-ranked treatment with a probability of 0.34. Statistical analysis revealed that despite exhibiting greater AEs, the above treatment did not differ significantly in this regard compared to FULV (HR=12.1, 95% CI 0.23-709.63), FOLFOX + B (HR=1.73, 95% CI 0.04-83.94), FOLFIRI + B (HR=2.19, 95% CI 0.05-113.31), FOLFIRI + cetuximab C(HR=1.44, 95% CI 0.03-77.82), FOLFOXIRI + cetuximab C (HR=1.96, 95% CI 0.04-106.33), FOLFOX +P (HR=1.3, 95% CI 0.03-65.86), FOLFIRI + P (HR=2.11, 95% CI 0.04-138.55), FOLFOXIRI + P (HR=0.75, 95% CI 0.02-42.08), FOLFOX + vanucizumab (HR=1.98, 95% CI 0.04-110.39), XELOX (HR=4.67, 95% CI 0.1-254.69), FOLFOX (HR=3.18, 95% CI 0.08-164.41), FOLFIRI (HR=3.18, 95% CI 0.08-199.23), FOLFOXIRI (HR=1.65, 95% CI 0.03-111.78), and FULV + B(HR=4.08, 95% CI 0.07-257.61). Compared to the FOLFOX + avelumab BAVENCIO + AdCEA vaccine treatment, greater but not significantly different AEs were noted for FOLFOXIRI + B (HR=0.89, 95% CI 0.02-44.25), FOLFOX + cetuximab C (HR=0.8, 95% CI 0.02-42.89), and FOLFOXIRI + B + A (HR=0.7, 95% CI 0.01-39). Of note, equally stronger AEs were observed for FOLFOXIRI + B +A and FOLFOXIRI + P.

#### 3.8.2 Subgroup grade ≥3 AEs analysis

**Figure 8:**
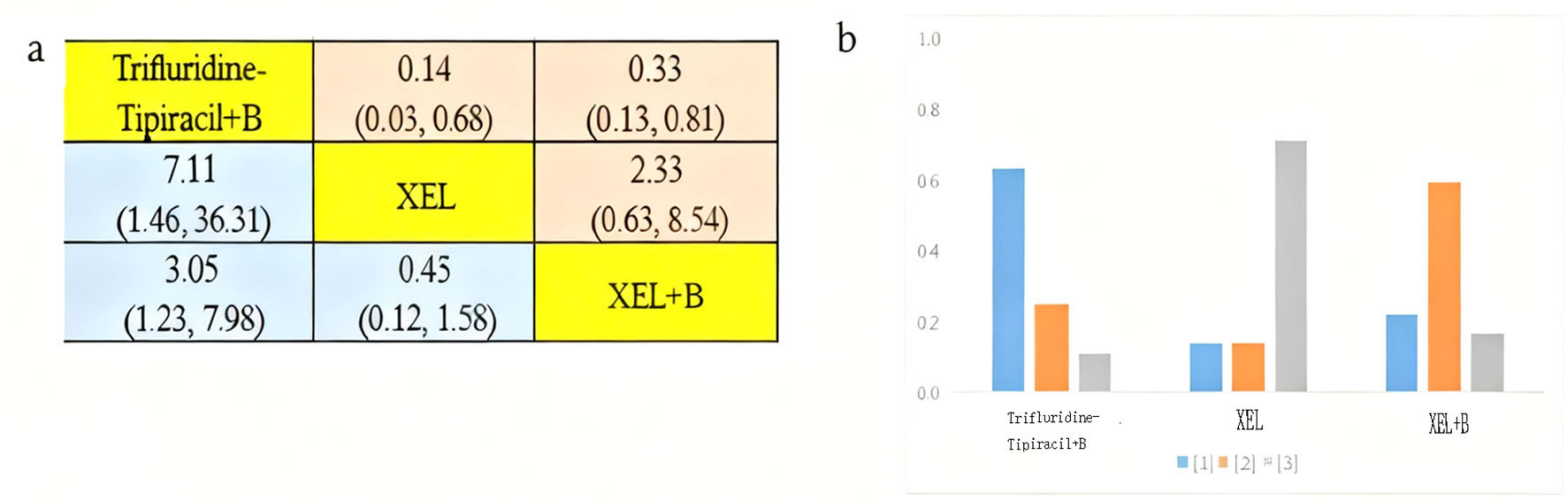
Incidence of grade ≥3 AEs in patients aged over 70 years. **(A)** Forest plot of grade ≥3 AEs; **(B)** Rank probability of grade ≥3 AEs.

The incidence of grade **≥**3 AEs was analyzed among 1289 patients aged over 70 years across three clinical trials involving three distinct treatment regimens: trifluridine-tipiracil + B Beva, XEL + B Beva, and XEL (**Figure 3H**). **Figure 8** presents a bar chart depicting the ranking probabilities of the incidence of grade **≥**3 AEs associated with each regimen. Trifluridine-tipiracil + B emerged as the top-ranked treatment, with a probability of 0.63, but despite exhibiting a higher incidence of AEs it did not differ from either XEL + B (HR=3.05, 95% CI 1.23-7.98) and XEL (HR=7.11, 95% CI 1.46-36.31). In summary, trifluridine-tipiracil + B demonstrated a higher incidence of grade **≥**3 adverse reactions among mCRC patients over 70 years of age.

### 3.9 SUCRA results

#### 3.9.1 PFS and OS for all patients

SUCRA analysis is utilized to ascertain the ranking probabilities for clinical treatments per safety and efficacy outcome. In this study, SUCRA scores were obtained for PFS data from 25 treatment regimens, OS data from 21 regimens, and grade ≥3 AEs data from 18 regimens (**Table 2**). According to the SUCRA ranking, FOLFOXIRI + B + A holds the highest likelihood of ranking first for PFS, with a probability of 97.0%. Similarly, FOLFOXIRI + B + A has the greatest potential (93%) to rank first in terms of OS benefit. Regarding treatment safety, FOLFOXIRI + B + A (86%) and FOLFOXIRI + P (85%) emerge as the most probable candidates for ranking first in terms of a higher incidence of grade ≥3 AEs. In summary, while FOLFOXIRI + B + A demonstrates superior PFS and OS for mCRC, it is associated with a correspondingly higher rate of grade ≥3 AEs.

**Table 2:** SUCRA ranking for effectiveness and safety of different treatment regimens.

Treatment	PFS		OS		Grade ≥3 AEs	
	SUCRAs (%)	Rank	SUCRAs (%)	Rank	SUCRAs (%)	Rank
FOLFOXIRI+B+A	0.97	1	0.93	1	0.86	1
FOLFOXIRI+B	0.88	2	0.81	3	0.81	4
FOLFOX+N	0.81	3	–	–	–	–
Pembrolizumab	0.78	4	0.62	8		–
FOLFOX+B	0.67	5	0.64	7	0.52	9
XELOX+B	0.66	6	0.39	13	–	–
SIRI+B	0.65	7	0.39	14	–	–
FOLFIRI+P	0.62	8	0.75	5	0.45	12
FOLFOX + Avelumab + AdCEA Vaccine	0.62	9	–	–	0.63	6
FOLFOX+V	0.62	10	–	–	0.47	11
FOLFOXIRI+P	0.56	11	0.78	4	0.85	2
FOLFOX+P	0.56	12	0.85	2	0.66	5
XELOX+B+C	0.55	13	0.25	17	–	–
FOLFOX+C	0.47	14	0.60	9	0.83	3
FOLFOXIRI+C	0.46	15	0.52	10	0.49	10
X	0.41	16	0.25	18	–	–
FULV+B	0.40	17	–	–	0.22	15
FOLFOXIRI	0.38	18	0.36	16	0.53	8
FOLFIRI+B	0.36	19	0.38	15	0.42	13
IROX	0.32	20	0.15	19	–	–
FOLFIRI+C	0.32	21	0.67	6	0.61	7
FOLFOX	0.20	22	0.43	12	0.26	14
XELOX	0.15	23	0.47	11	0.14	17
FOLFIRI	0.07	24	0.12	21	0.20	16
FULV	0.01	25	0.13	20	0.01	18

#### 3.9.2 Subgroup SUCRA analysis

##### 3.9.2.1 RAS/BRAF wild-type subgroup

The SUCRA method was employed to ascertain the ranking probabilities of 11 treatment approaches on the PFS and OS of mCRC patients in the RAS/BRAF wild-type subgroup (**Table 3**). According to SUCRA, FOLFOXIRI + C holds the highest likelihood of ranking first for PFS, with a probability of 77%. Similarly, FOLFOXIRI + C demonstrates also the greatest potential to rank first in terms of OS benefit, with a probability of 67%. In terms of efficacy, FOLFOXIRI + C emerges as the superior choice, exhibiting the best PFS and OS outcomes.

**Table 3:** SUCRA ranking for efficacy of different treatment regimens in the RAS/BRAF wild-type subgroup.

Treatment	PFS		OS	
	SUCRAs(%)	Rank	SUCRAs(%)	Rank
FOLFOXIRI+C	0.77	1	0.67	1
FOLFOX+C	0.69	2	0.67	2
FOLFIRI+P	0.64	3	0.61	4
FOLFOX+B	0.59	4	0.44	10
FOLFOXIRI+P	0.57	5	0.45	8
FOLFOX+P	0.54	6	0.64	3
FOLFOXIRI	0.40	7	–	–
FOLFIRI	0.37	8	0.49	6
FOLFIRI+B	0.37	9	0.28	10
FOLFIRI+C	0.33	10	0.47	7
FOLFOX	0.23	11	0.56	5

##### 3.9.2.2 Patients aged ≥70 years

SUCRA scores were also calculated to evaluate PFS, OS, and occurrence of grade ≥3 AEs across three treatment modalities in mCRC patients aged 70 years or more (**Table 4**). Trifluridine-tipiracil + B emerged as the highest-ranking treatment in terms of both PFS, with a probability of 92%, and OS, with a probability of 76%. However, it also exhibited the highest incidence of grade ≥3 AEs, ranking first in this category with a SUCRA value of 76%. Therefore, among elderly individuals (≥70 years old), trifluridine-tipiracil + B demonstrated superior efficacy, with the most optimal PFS and OS outcomes, albeit accompanied by the highest incidence of grade ≥3 AEs.

**Table 4:** SUCRA ranking for effectiveness and safety of different treatment regimens in patients ≥70 years of age.

Treatment	PFS		OS		Grade ≥3 AEs	
	SUCRAs(%)	Rank	SUCRAs(%)	Rank	SUCRAs(%)	Rank
Trifluridine-Tipiracil+B	0.92	1	0.76	1	0.76	1
XEL	0.03	3	0.22	3	0.22	3
XEL+B	0.55	2	0.52	2	0.52	2

## 4 Discussion

We conducted an NMA for clinical trials evaluating 30 first-line interventions, the first-line treatment for patients with mCRC is systemic therapy based on chemotherapeutic agents or combined targeted agents and immunotherapeutic agents. Chemotherapy drugs are fluorouracil-based and can be combined with other cytotoxic drugs oxaliplatin and/or irinotecan, and fluorouracil anticancer drugs are currently mainly 5-FU and capecitabine. In addition, the targeted drugs currently recommended for first-line treatment of mCRC are mainly bevacizumab and cetuximab. For immunotherapy, about 5% of patients with mCRC have high microsatellite instability (MSI-H) due to DNA mismatch repair (dMMR) deficiency, which makes them highly sensitive to immune checkpoint inhibitors (ICIs) treatment. However, most patients with mCRC have normal mismatch repair function (pMMR) and microsatellite stability (MSS) and are resistant to treatment with ICIs. Therefore, NCCN guidelines/ESMO guidelines/CSCO guidelines (78–80). preferentially recommend immune checkpoint inhibitors for first-line treatment regimens in patients with MSI-H/dMMR mCRC. In this paper, network meta-analysis confirmed that FOLFOXIRI + B + Atezo regimen could achieve survival benefit in terms of PFS and OS in both MSI-H/dMMR population and MSS/MSI-L/pMMR population, and FOLFOXIRI + B + A was the best first-line treatment compared with other regimens, but MSS/MSI-L/pMMR population had high limited immune score and/or high TMB. Among patients with RAS/BRAF wild type mCRC, FOLFOXIRI + C exhibited remarkable PFS and OS (81). In turn, in the subgroup of patients aged over 70 years, trifluorouridine-tipiracil + B demonstrated improved PFS and OS.

We incorporated all reported clinical trials involving first-line immunotherapy for mCRC, encompassing patients with dMMR/MSI-H in the KEYNOTE-177 study receiving pembrolizumab, patients with microsatellite stability in the METIMMOX trial treated with FOLFOX + N, and patients with mCRC administered FOLFOXIRI + B + A in the AtezoTRIBE trial. Within the KEYNOTE-177 study, pembrolizumab demonstrated a superior median PFS compared to chemotherapy. While the difference in survival rates was not statistically significant, a crossover between arms was observed, and pembrolizumab was associated with improved quality of life (54). Additionally, the CheckMate 142 trial revealed that the combination of nivolumab and ipilimumab as a second-line treatment exhibited efficacy, with favorable 5-year follow-up results (82). Based on these findings, immune checkpoint inhibitors have been established as a therapeutic option for dMMR/MSI-H mCRC.

Our research results indicate that the combination of FOLFOXIRI with bevacizumab and atezolizumab provides the best PFS and OS compared to simple chemotherapy regimens (FULV, XELOX, FOLFOX, FOLFIRI, IROX, FORFOXIRI) or targeted combination chemotherapy regimens (FULV, XELOX, FOLFOX, FOLFIRI, or FOLFOXIRI, combined with bevacizumab or EGFR antibodies, e.g. cetuximab and panitumumab). In particular, we found that FOLFOXIRI + B + A significantly improves PFS and OS compared to FOLFOXIRI + B. The results of the AtezoTRIBE study confirmed that compared to FOLFOXIRI + B, FOLFOXIRI +B +A significantly improved the PFS rate in unresectable and previously untreated mCRC patients, with good safety (53). An NMA incorporating the results of the AtezoTRIBE study has not yet been reported, but a NMA study reported by Wei et al. showed that FOLFOXIRI + B was significantly better than most other treatment options in terms of objective response rate (ORR), disease control rate (DCR), PFS, and OS (83). This combination is supported by studies such as TRIBE2, which emphasized the benefits of triple chemotherapy in improving response rates and potentially extending survival in certain patient populations (84). Our research results also support the use of intensive chemotherapy strategies combined with multiple biologic agents for the treatment of mCRC. This NMA also confirmed that FOLFOXIRI + B + A confers the best overall PFS and OS and is most likely to become the first-line treatment of choice for mCRC from the perspective of efficacy.

In subgroup analysis, Patients with KRAS or NRAS mutant tumors should not be treated with cetuximab alone or in combination with other anti-cancer drugs, as they have little chance of benefit and hence the exposure to toxicity and expense are not justified (85). FOLFOXIRI + C emerged as the treatment regimen of choice for RAS/BRAF wild-type patients in terms of PFS and OS. However, there was no statistical difference in the efficacy of FOLFOXIRI + C compared to FOLFOX + B, FOLFIRI + B, FOLFOX + C, FOLFIRI + C, FOLFOX + P, FOLFIRI + P, and FOLFOXIRI + P. Multiple studies have shown that in patients with unresectable mCRC, the first-line FOLFOXIRI regimen, whether combined with bevacizumab or not, has a higher ORR, complete tumor resection rate (R0), and median OS than the FOLFIRI or FOLFOX regimens (44, 74), indicating that the three-drug combination regimen is more effective than the two-drug combination regimen. However, no significant difference in resection rate and PFS outcomes were noted in the prospective, open-label, multicenter randomized controlled TRICE study (86), which evaluated patients who had not received first-line treatment and were allocated to either an experimental group receiving FOLFOXIRI (three-drug group) combined with cetuximab, or a control group receiving FOLFOX (two-drug group) combined with cetuximab. Whether combined with two-drug chemotherapy or three-drug chemotherapy, cetuximab as a conversion treatment regimen for patients with RAS/BRAF wild-type colorectal liver metastases have shown higher ORR and higher conversion rate to surgical resection. However, the risk of grade 3–4 neutropenia and diarrhea was relatively high in the experimental group. Therefore, considering safety, the two-drug chemotherapy combined with cetuximab regimen may be presently a more appropriate recommended regime (87).

Drawing upon the research findings from the SWOG S1406 and BEACON trials (82, 88), second-line or higher treatment recommendations primarily involve multi-target drug combination therapies, such as VIC (vemurafenib + irinotecan + cetuximab) or cetuximab combined with a BRAF inhibitor, optionally paired with a mitogen-activated protein kinase kinase (MEK) inhibitor (85). Nevertheless, despite improvements in clinical outcomes observed in patients with BRAFV600E-mutant mCRC who received a combination of BRAF inhibitors and EGFR and/or MEK inhibitors, response rates remain relatively low and lack sustained effectiveness.

According to FDA data, only 24% of patients participating in cancer drug clinical trials were 70 years of age or older, and most clinical trials excluded elderly cancer patients from enrollment. Normative clinical data is lacking for the treatment of elderly patients over 70 years of age. Moreover, the NCCN guidelines/ESMO guidelines do not mention the treatment of elderly patients for first-line treatment regimens for colon cancer, but the guidelines divide mCRC patients into those who are suitable for high-intensity treatment and those who are not suitable for high-intensity treatment, however, elderly patients become one of the important factors that are not suitable for high-intensity treatment. Therefore, the American Society of Clinical Oncology (ASCO) updated the Practical Guidelines for Vulnerability Assessment and Management of Elderly Patients Receiving Systemic Anticancer Therapy in 2023, emphasizing the core position of the Geriatric Assessment (GA) in the management of elderly cancer patients (80). Trifluorouridine-tipiracil is used in the NCCN guidelines/ESMO guidelines for the treatment of patients with mCRC who have previously received chemotherapy and targeted agents (Class IA), while the Chinese CSCO guidelines list it as first-line treatment not suitable for high-intensity treatment (Class IIB) and as previously received chemotherapy and targeted agents for the treatment of patients with mCRC (Class IA), therefore, this study confirmed thattrifluorouridine-tipiracil combined with bevacizumab regimen has certain advantages in PFS and OS for patients aged ≥70 years, which is consistent with the treatment regimen recommended by the CSCO guidelines (Class IIB), but large-scale clinical trials are still needed for validation.

In the context of mCRC treatment, safety remains paramount. When compared to other targeted chemotherapy regimens, the incidence of grade 3 or higher AEs is notably higher in the triple drug chemotherapy combination than in the dual drug chemotherapy combination. A systematic review, inclusive of a meta-analysis encompassing five randomized controlled trials (89), underscores this observation. Comparing the combination of bevacizumab with dual chemotherapy (FOLFIRI or FOLFOX) to triplet chemotherapy (FOLFOXIRI), it becomes evident that the likelihood of experiencing AEs such as diarrhea, neurotoxicity, and neutropenia is significantly elevated in the triple chemotherapy regimen. Our findings echo this trend, with our safety analysis revealing an 86% incidence of grade 3 AEs for the FOLFOXIRI + B+ A combination and an 85% incidence of AEs for the FOLFOXIRI + P combination. Overall, the occurrence of grade 3 and higher AEs is higher when chemotherapy is combined with targeted/immunotherapy than when chemotherapy is administered alone, but no new fatal treatment-related AEs were observed, and the patients tolerated well, therefore, the safety of this regimen was manageable (49).

Additionally, among patients with an average age exceeding 70 years, the incidence of AEs for the trifluridine-tipiracil + B regimen is particularly high, reaching 76%. However, the age-based SUNLIGHT trial showed that the incidence of AEs, including neutropenia, nausea, and anemia, was similar across age groups during treatment with trifluorouridine-tipiracil in combination with bevacizumab and was well tolerated. 96 Thus, despite the high ranking of Grade≥3AEs, the incidence of AEs was similar across age groups and well tolerated.

The reticulated meta-analysis of this study found no inconsistency in this study by performing inconsistency tests between direct and indirect evidence, which suggests that the results of this study are reliable. Although there are other similar reticular meta-analyses on the treatment of metastatic colorectal cancer, some studies only analyze chemotherapy regimens or chemotherapy combined with targeting, which has no guiding significance for chemotherapy combined with targeted drugs and immunotherapy regimens in clinical practice. There were also studies that did not limit whether the treatment regimen was first-line and did not stratify for age. This study is the first to perform a reticular meta-analysis of the efficacy of 30 first-line systemic treatment regimens for metastatic colorectal cancer, and includes chemotherapy combined with targeted and immunotherapy regimens, age subgroups and gene mutation subgroups, providing a reference for the selection of clinical treatment regimens. This approach ensures alignment between the chosen treatment and the molecular characteristics of the tumor, optimizing therapeutic efficacy while minimizing unnecessary toxicities (90). This study also has some limitations: First, because some of the patients included in the study did not screen the MSI-H/dMMR population or the MSS/pMMR population, no subgroup analysis was performed based on the MSI-H/dMMR or MSS/pMMR of the patients in this study. Second, some clinical trials failed to obtain the final data of OS and ≥ Grade 3 AEs, and some outcome measures could not be fully analyzed, so they were not included in this network meta-analysis, and the results may have some deviations. Third, some treatment regimens lack direct comparative studies, and the number of included studies and the total number of study subjects in each treatment regimen are inconsistent. Fourth, the results of this study may be compromised by the lack of unpublished literature, which may cause some poor information on the accuracy of our results.

There remains an urgent demand for direct comparative trials involving key treatment options, particularly those focusing on genetically stratified patient populations. Such studies are vital for enhancing current treatment guidelines and integrating novel therapeutic plans (91, 92). Furthermore, exploring long-term outcomes, such as quality of life and functional status post-treatment, will provide invaluable insights for guiding treatment decisions, not only extending lifespan but also enhancing quality of life (93, 94).

## 5 Conclusions

This analysis confirms the significant benefits of combining targeted therapy and immunotherapy with chemotherapy in first-line treatment of mCRC, tailored to genetic characteristics. It supports a shift towards more personalized and precise treatment strategies, with the potential to improve the prognosis of mCRC patients. The ongoing research and clinical practice updates based on new evidence will continue to impact the future of mCRC treatment. Furthermore, translational studies to identify biomarkers of sensitivity and resistance to different treatment options will help shaping more personalized therapeutic sequences.

## Data Availability

The original contributions presented in the study are included in the article/supplementary material. Further inquiries can be directed to the corresponding authors.

## Glossary

A: Atezolizumab
B: Bevacizumab
C: Cetuximab
FOLFIRI: Irinotecan + Leucovorin + 5-Fluorouracil
FOLFOX: 5-Fluorouracil + Oxaliplatin + Leucovorin
FOLFOXIRI: Oxaliplatin + Irinotecan + Leucovorin + 5-Fluorouracil
FULV: Fulvestrant
IROX: Irinotecan + Oxaliplatin
P: Pembrolizumab
SOX: S1 (Tegafur, Gimeracil and Oteracil Potassium Capsules) + Oxaliplatin
XELOX: Oxaliplatin + Capecitabine
